# *OsASN1* Plays a Critical Role in Asparagine-Dependent Rice Development

**DOI:** 10.3390/ijms20010130

**Published:** 2018-12-31

**Authors:** Le Luo, Ruyi Qin, Tao Liu, Ming Yu, Tingwen Yang, Guohua Xu

**Affiliations:** 1 State Key Laboratory of Crop Genetics and Germplasm Enhancement, Nanjing Agricultural University, Nanjing 210095, China; luole@njau.edu.cn (L.L.); 2017103122@njau.edu.cn (R.Q.); 2018103131@njau.edu.cn (T.L.); 2018103132@njau.edu.cn (M.Y.); 2017803194@njau.edu.cn (T.Y.); 2 MOA Key Laboratory of Plant Nutrition and Fertilization in Lower-Middle Reaches of the Yangtze River, Nanjing Agricultural University, Nanjing 210095, China

**Keywords:** rice, asparagine, tiller, nitrogen

## Abstract

Asparagine is one of the important amino acids for long-distance transport of nitrogen (N) in plants. However, little is known about the effect of asparagine on plant development, especially in crops. Here, a new T-DNA insertion mutant, *asparagine synthetase 1* (*asn1*), was isolated and showed a different plant height, root length, and tiller number compared with wild type (WT). In *asn1*, the amount of asparagine decreased sharply while the total nitrogen (N) absorption was not influenced. In later stages, *asn1* showed reduced tiller number, which resulted in suppressed tiller bud outgrowth. The relative expression of many genes involved in the asparagine metabolic pathways declined in accordance with the decreased amino acid concentration. The CRISPR/Cas9 mutant lines of *OsASN1* showed similar phenotype with *asn1*. These results suggest that *OsASN1* is involved in the regulation of rice development and is specific for tiller outgrowth.

## 1. Introduction

Nitrogen (N) is one of the most important macro nutrients for plant growth and development. N is absorbed by plant roots mainly in two forms: ammonium and nitrate. Some of the nitrate is directly used by root, and some is reduced to ammonium. Ammonium was assimilated to glutamine by glutamine synthetase and glutamine-2-oxoglutarate aminotransferase and transported to the shoot. However, some of the glutamine could be synthesized to asparagine and other amino acids or other compounds containing N [1].

The most important forms of N transport from root to shoot is glutamine and asparagine. In rice xylem and phloem sap, glutamine is the most abundant amino acid for N transportation, the second is asparagine [2,3]. Glutamine synthetase (GS) catalyzes ATP-dependent conversion of glutamate into glutamine using ammonium [4,5]. There are two forms of GS, named cytosolic GS1 and chloroplastic GS2 [6]. There are multiple genes that belong to GS1 and are located in different tissues that play different roles in N assimilation [7,8]. GS2, which is localized in mitochondria or chloroplast, is related to the ammonia assimilation from nitrate reduction or photorespiration [9,10].

Other than glutamine, asparagine is the major N form in both phloem and xylem sap [2,11,12,13]. Asparagine has a high concentration of C/N, and is more stable compared with other amide compounds. Additionally, asparagine exhibits better solubility and mobility. The function of asparagine is not only as a N supply from root to shoot, but is also related to the re-localization from senescence organs to growing leaves and developing seeds [3,14]. There are two forms of asparagine synthetases (ASN), named asparagine synthetase-A (ASN-A) and asparagine synthetase-B (ASN-B). Only ASN-B is found in plants and regulated by light and metabolites involved in plant development, such as seed development and vegetative organ growth [15]. In Arabidopsis, there are three genes that encode for asparagine synthetase, which belong to two groups [15]. *AtASN1* belongs to Class I, and *AtASN2* and *AtASN3* belong to Class II [15]. *AtASN1* and *AtASN3* showed quite different expression patterns compared with *AtASN2*, and these genes are induced by quite different stimuli [15]. The analysis of *ASN* mutants indicated that *AtASN2* plays a role in the primary N assimilation for N storage and export [16,17]. Decades ago, asparagine synthetase was analyzed in rice, and the expression pattern and function were investigated [18,19]. In rice, there are two genes encoding asparagine synthetase, *OsASN1* and *OsASN2* [13]. *OsASN1* and *OsASN2* showed different expression patterns and response to ammonium. The analysis of knock out mutants showed that *OsASN1* is responsible for the synthesis of asparagine in rice root [13].

In this study, a T-DNA insertion mutant was isolated, and phenotypic analysis showed significantly reduced growth compared with WT, especially in tiller numbers. It was found that the reduced tiller number was caused by the changing of asparagine concentration, where as N absorption was not influenced. These results indicate that asparagine is important for plant development.

## 2. Results

### 2.1. Screening of a New T-DNA Insertion Mutant with Growth Defects

**Figure 1:**
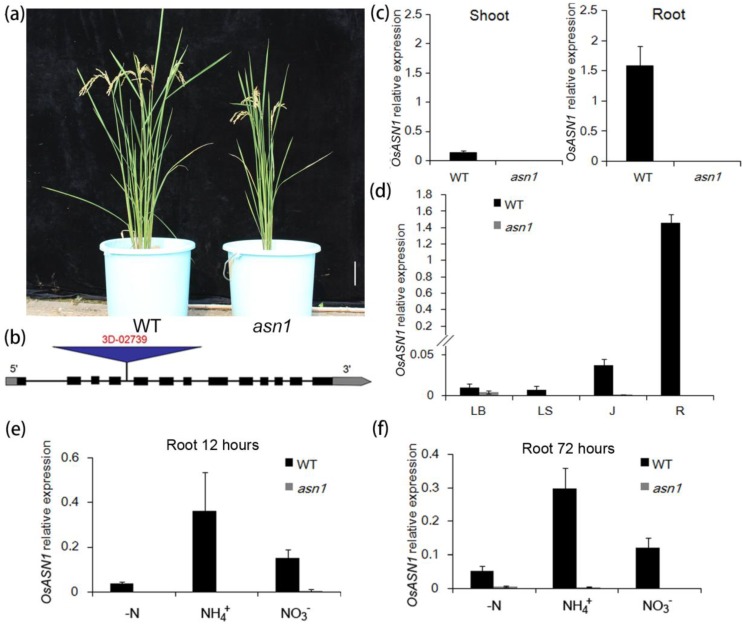
Isolation of a T-DNA insertion mutant. (**a**) Wild type (WT) and T-DNA insertion mutant *asn1*; (**b**) position of T-DNA insertion into *OsASN1* genome; (**c**) relative expression of *OsASN1* in shoot and root of seven-day-old seedling; (**d**) relative expression of *OsASN1* in leaf blade (LB), leaf sheath (LS), junction (J), and root (R) of 21-day-old seedling; (**e**) relative expression of *OsASN1* in the root 12 hours after nitrogen deficient (–N), 2.5 mM NH_4_^+^, and 2.5 mM NO_3_^−^ treatments; (**f**) relative expression of *OsASN1* in root 72 hours after nitrogen deficient treatment, 2.5 mM NH_4_^+^, and 2.5 mM NO_3_^−^ treatment.The values are the transcriptional expression level of *OsASN1* to *OsActin* (internal standard control) by quantitative Polymerase Chain Reaction (qPCR). The scale baris 10 cm. Data are mean ± S.D. (*n* = 3).

For the aim of isolating mutants with tiller number defects, several T-DNA insertion lines were ordered for phenotypic observation. One line that has an insertion in the fourth intron of *asparagine synthetase 1* (*ASN1*) showed strong growth defects compared with wild type (WT) (Figure 1a,b). To confirm that *OsASN1* was a knockdown mutant, primers that bind to the region of the T-DNA insertion were designed, and the RNA of *OsASN1* was detected. In the seven-day old plants, *OsASN1* was not detected in both shoot and root (Figure 1c). In the 21-day-old plants, *OsASN1* transcripts were hardly detected in leaf blade, leaf sheath, and junction, and even highly expressed tissue and root (Figure 1d). The expression of *OsASN1* was further detected in the highly expressed tissue under treatment with different N forms. The expression of *OsASN1* could not be detected even in highly induced conditions, such as ammonium treatment in root (Figure 1e,f). To confirm the insertion number, southern blot was performed (Figure S1). Only one band could be detected in *asn1* plant after DNA was digested by restriction enzyme Hind III and BamH I, while in WT there was no band detected. These all suggest that *asn1* was suitable for the analysis of *ASN1* function.

### 2.2. asn1 Shows Growth Defects at Both Early and Late Stages

**Figure 2:**
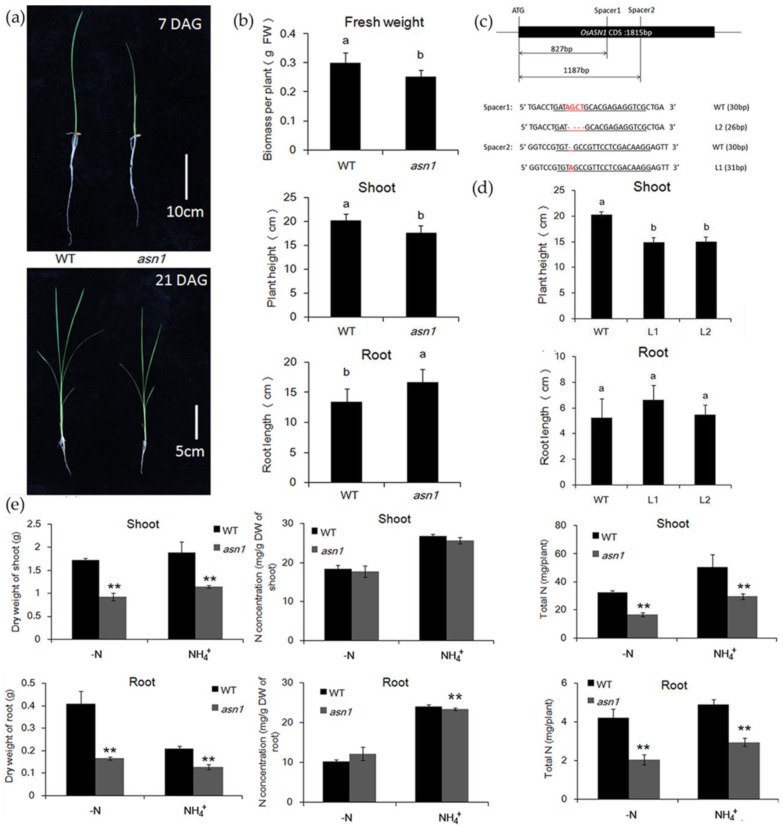
Phenotypic analysis of *asn1*. (**a**) Seven- and 21-day-old seedlings of wild type (WT) and *asn1*, DAG, days after germination; (**b**) fresh weight (FW), shoot, and root length of WT and *asn1* when fifth leaf fully expanded, data are mean ± SD (*n* ≥ 8),means with different letters were significantly different; (**c**) information of mutants generated by CRISPR/Cas9 system, the underline indicates the position of spacers and the red color highlights the mutation positions; (**d**) root length and height of WT and line 1 (L1), line 2 (L2). Data are mean ± SD (*n* ≥ 3), means with different letters were significantly different; (**e**) dry weight (DW), nitrogen concentration, and total nitrogen of WT and *asn1,* with and without 2.5 mM NH_4_^+^, for three weeks after one-month pre-culture. Data are means ± SD (*n* = 5). Significant differences were determined by ANOVA (** *p* < 0.01).

To analyze the different phenotypes between WT and *asn1*, the growth after germination was observed. The plant of *asn1* was smaller, compared with WT, seven days after germination (Figure 2a). Mutants were also generated using the CRIPR/cas9 system and the early stage phenotype was also analyzed (Figure 2c,d). At later stages, the difference still exists. When the fifth leaf fully expanded, the fresh weight and plant height were significantly reduced compared with WT, and the root length was increased (Figure 2a,b). Both the shoot and root dry weights of *asn1* were significantly decreased compared to WT, with and without 2.5 mM ammonium (Figure 2e). When measuring the N concentration, only roots under N-deficient conditions showed a significant decrease compared with WT (Figure 2e). In the *asn1* mutant, the growth was suppressed compared with WT, while the N concentration was not significantly influenced, which indicated that the total N utility was not influenced.

### 2.3. The Asparagine Metabolism Pathway Is Strongly Influenced in asn1 Mutant

**Figure 3:**
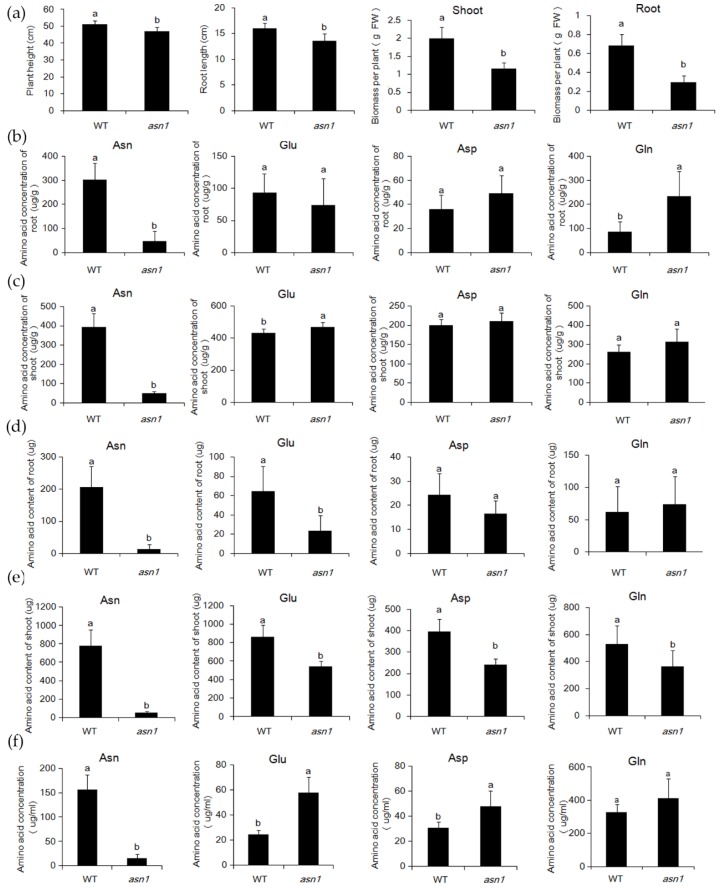
Comparison of asparagine, glutamate, aspartate, and glutamine in shoot, root and xylem sap of WT and *asn1*. (**a**) Plant height, root length, and biomass of WT and *asn1* after treatment with 2.5 mM NH_4_^+^for two weeks; FW, fresh weight; (**b**) concentration of asparagine (Asn), glutamate (Glu), aspartate (Asp), and glutamine (Gln) in the root of WT and *asn1*; (**c**) concentration of Asn, Glu, Asp, and Gln in the shoot of WT and *asn1*; (**d**) content of Asn, Glu, Asp, and Gln in the root of WT and *asn1*; (**e**) content of Asn, Glu, Asp, and Gln in the shoot of WT and *asn1*; (**f**) concentration of Asn, Glu, Asp, and Gln in xylem sap of WT and *asn1* treatment with 2.5 mM NH_4_^+^ for four weeks. Data are means ± SD (*n* ≥ 5). Means with different letters were significantly different.

**Figure 4:**
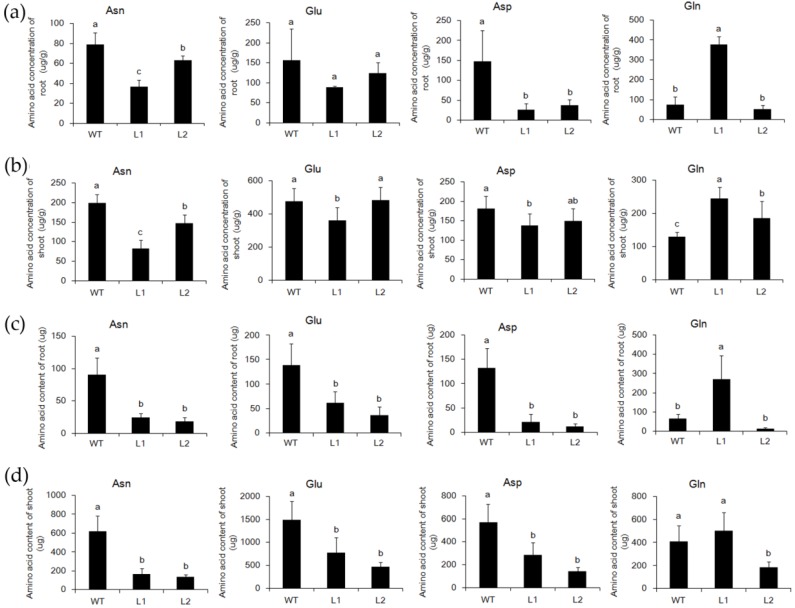
Comparison of asparagine, glutamate, aspartate, and glutamine in shoot and root of WT and L1, L2. (**a**) Concentration of asparagine (Asn), glutamate (Glu), aspartate (Asp), and glutamine (Gln) in the root of WT and L1, L2; (**b**) concentration of Asn, Glu, Asp, and Gln in the shoot of WT and L1, L2; (**c**) content of Asn, Glu, Asp, and Gln in the root of WT and L1, L2; (**d**) content of Asn, Glu, Asp, and Gln in the shoot of WT and L1, L2. Data are mean ± SD (*n* ≥ 6). Means with different letters are significantly different.

As the concentration of N was not significantly influenced, to investigate whether the loss of function of *ASN1* influenced the asparagine metabolism or not, the amount of asparagine, aspartate, glutamine, and glutamate were measured in WTand *asn1*. The plants were germinated in 1/2 Murashige and Skoog (MS), and transferred to 2.5 mM (NH_4_)_2_SO_4_ for two weeks. The plant of *asn1* was smaller compared with WT, showing reduced plant height, root length, and biomass (Figure 3a). In root, the concentration of asparagine was reduced to a sixth of WT (Figure 3b), and the concentration of glutamine was increased to two-fold compared to WT, which was probably caused by the reduced activity of *ASN1* protein, and the substrate remained (Figure 3b). The concentration of glutamate and aspartate was not significantly changed (Figure 3b). In the shoot, the concentration of all four amino acids increased (Figure 3c). The concentration of asparagine significantly decreased, which resulted in the loss of function of *OsASN1* (Figure 3c). The concentrations of other amino acids in *asn1* were not significantly different in WT (Figure 3c). The biomass of *asn1* shoot reduced to half of WT (Figure 3a), and the biomass of *asn1* root reduced to a third of WT (Figure 3a). The total amount of amino acid was significantly changed, which was partially caused by the reduced biomass of *asn1* (Figure 3d,e). As glutamine and asparagine are the two main forms of N transported from the root to shoot, the xylem sap was collected and the concentration of amino acids was measured. The concentration of asparagine significantly decreased, while no significant difference was exhibited in the concentration of glutamine (Figure 3f). In the CRISPR/Cas9 mutant lines L1 and L2, the same tendency was observed (Figure 4a,d). The asparagine metabolism was strongly influenced when *OsASN1* function was lost.

### 2.4. OsASN1 Influences the Tiller Phenotype

**Figure 5:**
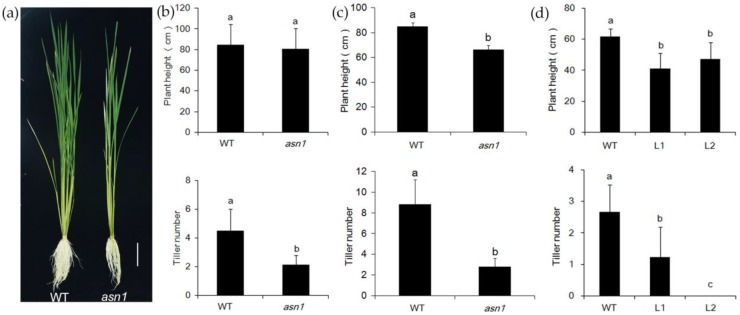
Phenotypic analysis of WT and *asn1* at the later stage. (**a**) WT and *asn1* treated with 2.5 mM NH_4_^+^for 60 days; (**b**) plant height and tiller number of plants in (**a**); (**c**)plant height and tiller number of plants in paddy field at heading stage; (**d**) plant height and tiller number of WT and L1, L2. The scale bar is 10 cm. Data are mean ± SD (*n* ≥ 8). Means with different letters are significantly different.

**Figure 6:**
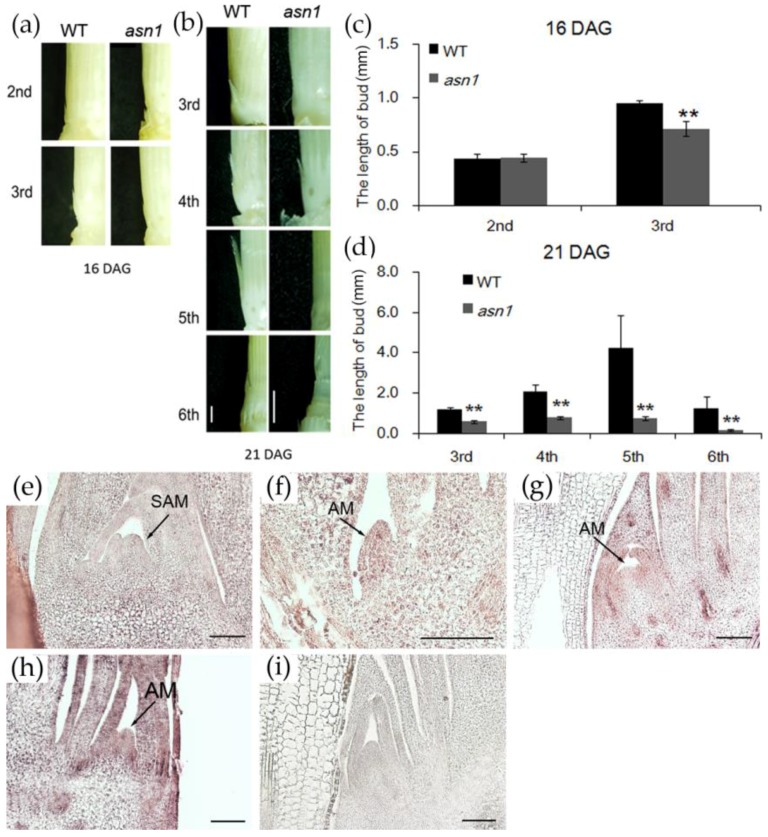
Comparison of tiller bud outgrowth in WT and *asn1*. (**a**) Tiller buds in the axil of second and third oldest leaves, 16 days after germination (DAG); (**b**) tiller buds in the axil of third, fourth, fifth, and sixth oldest leaves, 21 DAG; (**c**)the length of buds in (**a**); (**d**) the length of buds in (**b**). Data are means ± SD (*n* ≥ 8). Significant differences were determined by ANOVA (** *p* < 0.01). The scale bar is 1 mm; (**e**) expression pattern of *OsASN1* by RNA in situ hybridization, detection of *OsASN1* transcripts in shoot apical meristem (SAM); (**f**–**h**) detection of *OsASN1* transcripts in axillary meristem (AM) from early to late stages; (**i**) RNA in situ with sense probe. The scale bar is 100 μm.

At later stages, in hydroponic culture with 2.5 mM NH_4_^+^ supply, the difference in plant height decreased, but the number of tillers became significantly different (Figure 5a). In the growth conditions of this experiment, the tiller number of *asn1* was decreased to half of WT (Figure 5b). In the paddy field, at flowering time, both plant height and tiller number of *asn1* decreased significantly (Figure 5c). In the CRISPR/Cas9 mutant lines L1 and L2, the plant and tiller number of L1 and L2were significantly lower compared with WT (Figure 5d). The constant change of tiller number indicated that *ASN1* probably influenced the tiller development. To investigate whether the change of tiller number was influenced by the disordered bud initiation or the suppressed outgrowth, the buds of *asn1* and WT were observed at 16 and 21 days after germination, when the fifth and sixth leaves fully expanded (Figure 6a,b). Sixteen days after germination, the fifth leaf of plants fully expanded, and the tiller buds at the second and third leaves were observed (Figure 6a). There was no significant difference in the length of second tiller buds between WT and *asn1* (Figure 6c). The third bud was significantly shorter compared with WT (Figure 6c). When the sixth leaf fully expanded, tiller buds at the axil from the third to sixth leaf were observed (Figure 6b). The buds of *asn1* were significantly shorter compared with WT (Figure 6d). After the buds were initiated, the growth rate was suppressed in *asn1*. In the CRISPR/Cas9 mutant lines L1 and L2, the number of buds were significantly lower compared with WT (Figure 6e). The expression pattern of *OsASN1* was analyzed by in situ hybridization. The expression of *OsASN1* was hardly detected in the shoot apical meristem (SAM) (Figure 6f). In the tiller buds, when the buds grew, the expression of *OsASN1* seemed much stronger (Figure 6g–i). Tiller elongation was influenced in *asn1* plants.

### 2.5. ASN1 Does Not Influence the N Absorption

**Figure 7:**
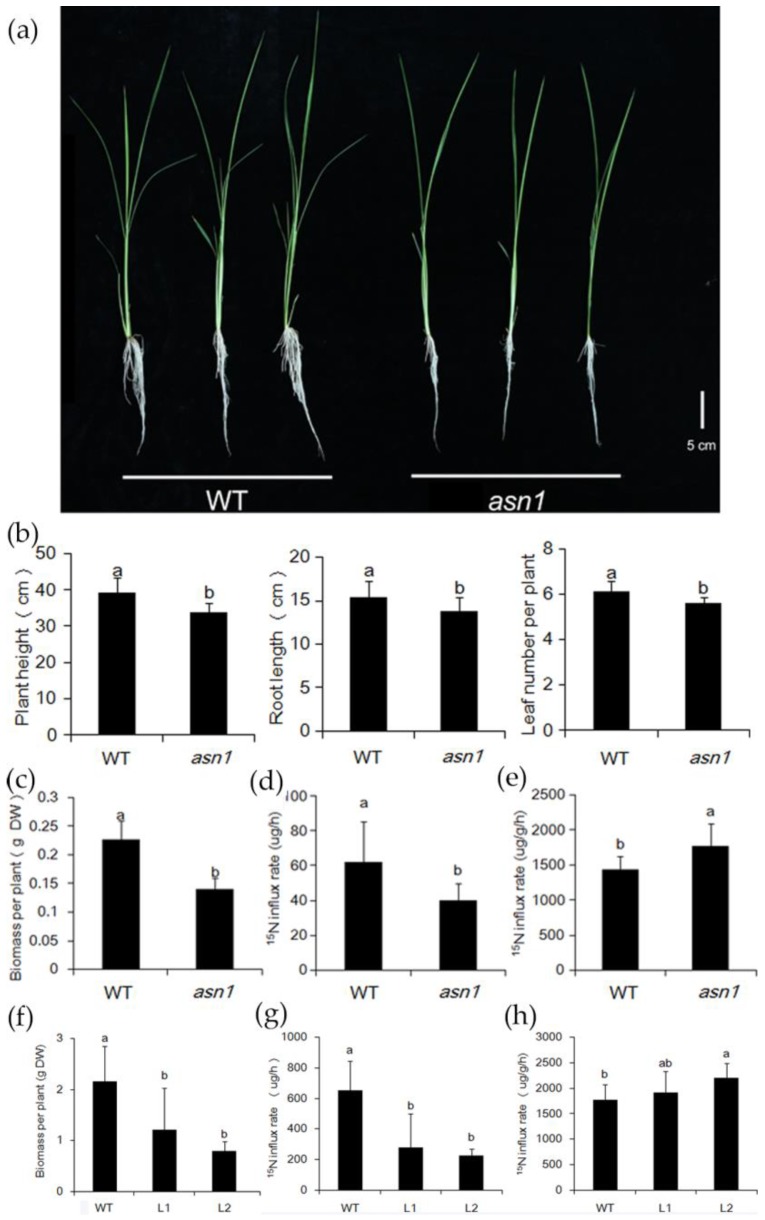
^15^N-NH_4_^+^ influx rate of WT and *asn1*. (**a**) WT and *asn1* were cultured for 20 days; (**b**) plant height, root length, and leaf number of WT and *asn1*; (**c**) biomass of WT and *asn1*; DW, dry weight; (**d**) ^15^N influx rate of WT and *asn1* per plant; (**e**) ^15^N influx rate of WT and *asn1* per unit root weight. Data are mean ± SD (*n* ≥ 9). Means with different letters are significantly different; (**f**) biomass of WT and L1, L2; (**g**) ^15^N influx rate of WT and L1, L2 per plant; (**h**) ^15^N influx rate of WT and L1, L2 per unit root weight. Data are mean ± SD (*n* ≥ 4). Means with different letters are significantly different.

To investigate whether the suppressed growth was influenced by the difference of the N influx rate, short-term ammonium absorption was assessed by transferring the plants to 2.5 mM ^15^NH_4_^+^ for five minutes. The plant height, root length, and leaf number per plant were shorter in *asn1* compared with WT, but the difference was not significant (Figure 7a,b). The ^15^NH_4_^+^ influx rate was lower in *asn1*, which was probably caused by the small biomass (Figure 7c,d). When calculated with the biomass, the ^15^NH_4_^+^ influx rate was increased in *asn1* (Figure 7e). In the CRISPR/Cas9 mutant lines L1 and L2, the same tendency was observed (Figure 7f–h). This indicated that the N absorption was not the cause of the mutant phenotype.

### 2.6. ASN1 Metabolism Is Significantly Influenced Which Caused the Phenotype Difference

**Figure 8:**
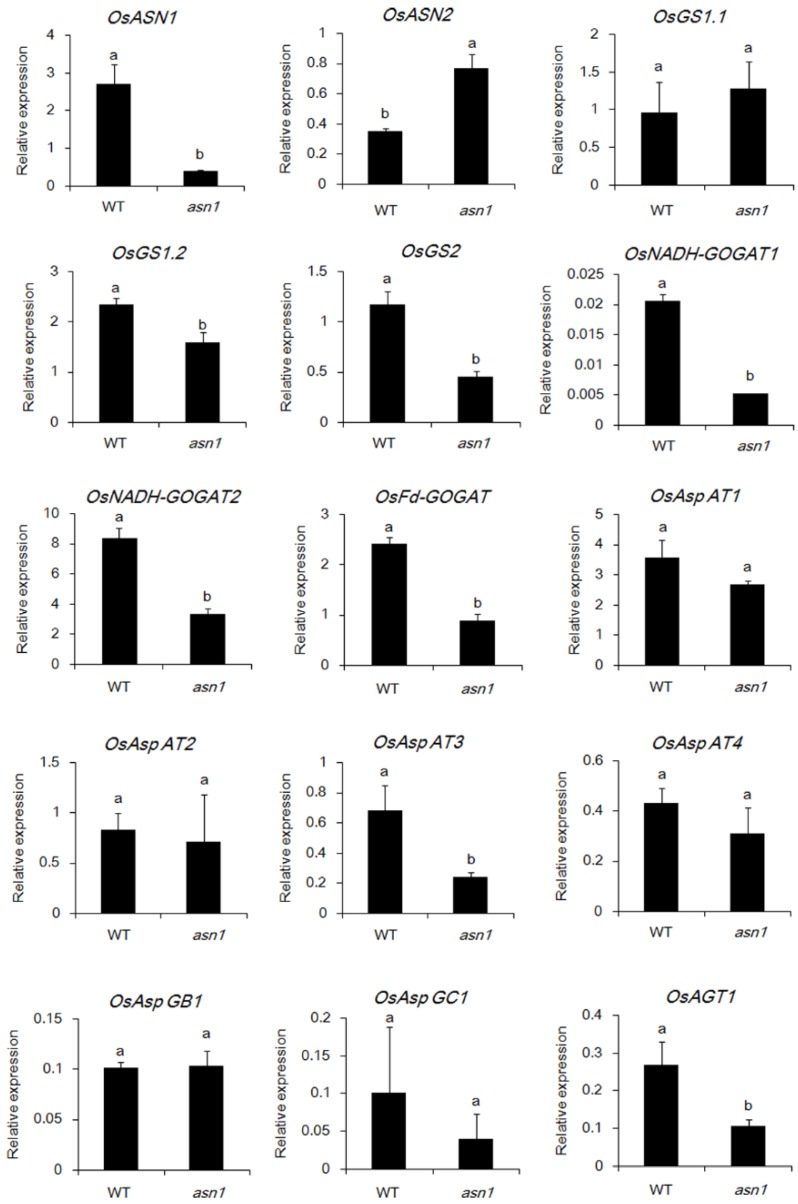
Relative expression of genes involved in the asparagine metabolic pathway. Genes involved in the asparagine metabolic pathway were listed as follows: *OsASN1*(*Os03g0291500*); *OsASN2*(*Os06g0265000*); *OsGS1.1*(*Os02g0735200*); *OsGS1.2*(*Os03g0223400*); *OsGS2*(*Os02g0701300*); *OsNADH-GOGAT1*(*Os01g0681900*); *OsNADH-GOGAT2*(*Os05g0555600*); *OsFd-GOGAT*(*Os07g0658400*); *OsAspAT1*(*Os01g0760600*); *OsAspAT2*(*Os02g0797500*); *OsAspAT3*(*Os02g0236000*); *OsAspAT4*(*Os06g0548000*); *OsAspGB1*(*Os04g0682500*); *OsAspGC1*(*Os04g0549300*); and *OsAGT1* (*Os08g0502700*). Plants were grown with normal nitrogen supply for one month and the root-shoot junctions were sampled. The values are the transcriptional expression level of upper listed genes to *OsActin* (internal standard control) by quantitative PCR. Data are mean ± SD (*n* = 3). Means with different letters are significantly different.

To investigate how *OsASN1* influences the phenotype of plants, especially the tiller outgrowth, the expression of genes involved in the asparagine metabolic pathway in the shoot of WT and *asn1* plants was analyzed (Figure 8; Table S1). The relative expression of *OsASN1* was very low in *asn1*, which was the same as detected before. Relative expression of *OsASN2* was increased, probably caused by the compensation mechanism of plants. Although the relative expression of *OsGS1.1* was not significantly changed in WT and *asn1*, the genes involved in glutamine and glutamate transformation decreased. For the decreased amount of asparagine, the enzymes in the downstream reactions, such as *OsAspATs* and *OsAspGs*, all showed decreased expression. These results indicate that loss of function of *OsASN1* caused the metabolic decline related to asparagine, which caused the affected development of plants, especially the tiller outgrowth.

## 3. Discussion

In this study, the T-DNA insertion mutant of *OsASN1* was analyzed in detail, and CRISPR/Cas9 lines of this gene were also constructed to confirm the phenotype. In the mutants, the expression of *OsASN1* was blocked and showed severe defects on the development. The generation of asparagine was strongly affected, which resulted in the sharply reduced amount of asparagine in both roots and shoots. The development of the *OsASN1* mutants, especially the development of tiller buds was influenced, suggesting that OsASN1 mediated asparagine metabolism pathway plays a critical role in tiller development in rice.

### 3.1. OsASN1 Influence the Development of Plants

In *asn1* mutants, the plant height and root length were different compared with WT. The tiller number was significantly changed, which indicated that asparagine was important for the tiller development. Our data indicated that the tiller formation was not influenced, whereas the tiller elongation was influenced (Figure 5). Recently, there are several reports about the influence of tillers by nutrients. N and phosphate are important for the regulation of tiller outgrowth [20,21]. Under N-deficient conditions, the tiller bud’s outgrowth was inhibited, and the cell division was ceased [20]. When decreasing Pi concentration, the levels of 2′-*epi*-5-deoxystrigol (*epi*-5DS) were increased and caused the suppression of tiller outgrowth [21]. The altered expression of *nitrate transporter 1/peptide transporter* family (*NPF*) 7.7 influenced the shoot branching number, which was accompanied by the changed amino acid concentration [22]. Another two genes, *NPF 7.2* and *NPF7.3*, in the same family, were also reported to influence the tiller number of mutant and overexpression plants [23,24]. In rice, there are hundreds of amino acid transporters. Recently the regulation roles of amino acids and tiller growth started to be revealed. Blocking *amino acid transporter3* (*AAP3*) enhances the grain yield due to the release of bud outgrowth, and over expressing *AAP3* resulted in the enriched amount of amino acids and inhibited bud outgrowth [25]. Other than these, the function of cytosolic *GS1;2* in plant development was analyzed in detail [12,13,26], in addition to its involvement in the primary assimilation of ammonium in rice root, it is also important for the tiller bud outgrowth by regulating the N-dependent biosynthesis of cytokinin. Our present results indicate that *OsASN1* is also important for tiller outgrowth.

### 3.2. OsASN1 Is a Gene with Multiple Functions

Asparagine synthetase was discovered long ago, and from prokaryotes to eukaryotes, the gene and protein structure was examined. The expression pattern was studied and discovered to be regulated by light, dark, sugars, and metabolites, as well as the developmental processes [15,27]. In rice and Arabidopsis, several studies based on T-DNA insertion mutants revealed the function of *ASN* genes. In Arabidopsis, *AtASN2* was first found responsible for ammonium metabolism and important for the N assimilation and export during vegetative growth [17,28]. In the transgenic Arabidopsis with *AtASN1* driven by the 35S promoter, the amount of asparagine was increased in the phloem, and the amino acid content was increased in seeds [29]. Whereas the T-DNA insertion mutant of *AtASN1* showed defect seed development, and metabolite profiles revealed the carbon and N partitioning to generate energy via the tricarboxylic acid cycle, GABA shunt, and phosphorylated serine synthetic pathway [30]. In rice, the tissue localization and NH_4_^+^ response of *OsASN1* and *OsASN2* was investigated, and it was shown that *OsASN1* and *OsASN2* probably play different roles. *OsASN1* is responsible for the biosynthesis of asparagine coupled with the primary assimilation of NH_4_^+^ in rice roots and *OsASN2*, which is probably related to the long-distance transport of asparagine [13]. Here, three allelic mutants of *OsASN1*, including one T-DNA insertion line (*asn1*) and two CRISPR/Cas9 lines (L1 and L2), were used, which gives new information of *OsASN1* function in the shoot part. *OsASN1* was not only expressed in root, but was also expressed in the tillers (Figure 6). In *OsASN1* mutants, the amount of asparagine sharply decreased, which resulted in the suppressed growth of tiller buds (Figure 5 and Figure 6). This was not caused by the N absorption, which was confirmed by ^15^N absorption experiment (Figure 7). In a recent paper, it was reported that *OsASN1* was less affective for the tiller outgrowth in rice [31]. This inconsistent result may have been caused by the use of different mutants between these two studies. Our results, based on three allelic mutants of *OsASN1*, strongly support the regulatory role of OsASN1 in tiller development.

### 3.3. Lack of Asparagine Suppressed the Cell Division

In the former research, it was found that N deficiency suppressed the tiller outgrowth [20]. Here, asparagine was also an important factor for tiller outgrowth. The mechanism of asparagine on this aspect was unknown. However, in other species, asparagine synthetase was studied broadly. In the research of tumor cells, asparagine was found to be an important regulator of amino acid homeostasis, anabolic metabolism, and proliferation [32], and the function of asparagine synthetase was confirmed to suppress cell proliferation and inhibit tumor growth in gastric cancer cells, human melanomacells, and epidermoid carcinoma cells [33,34]. The flow cytometry assay showed that ASNS silencing arrested cell cycle progression at G0/G1 phase, and probably through regulating the expression of cell cycle molecules, such as CDK2 and Cyclin E1, as shown by quantitative real-time PCR [35]. These all shed light on the research of asparagine synthetase in plants. It would be valuable to study the relationship between asparagine and cell cycle progression in plants.

## 4. Materials and Methods 

### 4.1. Plant Materials and Growth Conditions

A T-DNA insertion mutant (3D-02739) was obtained from POSTECH in Yongin, Korea, T-DNA insertion homozygotes were identified by two rounds of PCR. The first round of PCR was used to identify the presence or absence of T-DNA insertion, and amplification was performed using a target gene fragment primer and a specific T-DNA fragment primer (Left primer 5′-3′: ATTCTTCACGTCTCTGCTGT; General Tail 5′-3′: AACGCTGATCAATTCCACAG).The second round of PCR was used to identify whether it was homozygous (Left primer 5′-3′: ATTCTTCACGTCTCTGCTGT; Right primer 5′-3′: TTTGCCTTACGAATTCTGAT).

The experiment thatused the T-DNA insertion mutant wasnamed *asn1*, in the background of rice variety ‘Hwayoung’ (Oryza sativa cv. Hwayoung). The rice seeds were sterilely sterilized, then transferred to 1/2 Murashige and Skoog medium for seedlings. After one week, they were transplanted to the tank (tank size: 35 cm × 24 cm × 13 cm), 20 seedlings were planted in a tank. The nutrient solution was replaced every 2 days. The nutrient solution was modified by the International Rice Research Institute (IRRI) (1.25 mM NH_4_NO_3_ or (NH_4_)_2_SO_4_ or Ca(NO_3_)_2_, 0.3 mM KH_2_PO_4_, 1 mM K_2_SO_4_, 1 mM CaCl_2_, 1 mM MgSO_4_, 0.5 mM Na_2_SiO_3_, 20 μM EDTA-Fe, 9 μM MnCl_2_, 20 μM H_3_BO_3_, 0.77 μM ZnSO_4_, 0.32 μM CuSO_4_, and 0.39 μM Na_2_MoO_4_, with a pH of 5.7). The culture conditions were 30 °C under light conditions, 22 °C under dark conditions, 16 h light, 8 h darkness, and the relative humidity was 70%. 

### 4.2. Generation of OsASN1 CRSPR/CAS9 Mutants

For gene mutation with the CRISPR/Cas9 system, two gene specific spacers (spacer 1: cctgatagctgcacgagaggtcg; spacer 2: ccgtgtgccgttcctcgacaagg) residing in exons were selected from the library provided [36]. These spacers were ligated to the intermediate vector pOs-sgRNA via *BsaI* and then introduced into pH-Ubi-cas9-7 through the use of GATEWAY technology [36]. The constructs were transformed into mature embryos developed from seeds of wildtype (WT) rice plants (cv. Nipponbare) via *Agrobacterium tumefaciens*-mediated transformation, as previously described [37].

### 4.3. RNA Extraction and Quantitative PCR Analysis

Total RNA was extracted using Trizol reagent (Life technologies, carisbad, CA, USA), and cDNA was synthesized by reverse transcription using PrimeScript^TM^ RT reagent kit (Takara, Shiga, Japan). Real-Time PCR uses the AceQ^®^ qPCR SYBR^®^ Green Master Mix kit (Vazyme, Nanjing, China). The reaction system was 0.5 μL cDNA (10 ng·μL^−1^ total RNA), 5 μL SYBR Premix Ex Taq (2×), 0.2 μL ROX Reference DyeII (50×), 0.2 μL left and right primer (10 μmol·L^−1^), 3.9 μL ddH_2_O; and the reaction volume was 10 μL. The amplification reactions were performed on a Thermo Lifetech ABI QuantStudio^TM^ 6 Flex system (Life Technologies, Carisbad, CA, USA) with following steps: 95 °C for 30 s; 95 °C for 5 s; 60 °C for 30 s, 40 cycles; 95 °C for 15 s; 60 °C for 60 s; 95 °C for 15 s. After the reaction was completed, a dissolution curve was performed to detect primer specificity. All the primers used for the qRT-PCR analysis of genes are listed in Table S1.

### 4.4. Analysis of Total N Concentration

Seeds were germinated with 1/2 Murashige and Skoog medium, then they were transferred to 2.5 mM ammonium nitrate nutrient solution for 4 weeks, and then transferred to 0 and 2.5 mM NH_4_^+^ nutrient solution for 3 weeks. The plants were taken out and quickly placed in an oven at 105 °C for 30 min. The shoot and root of the plants were separated and placed in an oven at 70 °C for 1 week. Then the weight of the shoot and the root was measured. Approximately 0.05 g of the plant dry sample was loaded into a digestion tube, and the digestion was carried out by the H_2_SO_4_-H_2_O_2_ method, and the total N content was determined using a flow analyzer (AA3, BranLuebbe, Norderstedt, Germany).

### 4.5. Analysis of Amino Acids Concentration

The samples were prepared as follows: 2 g of frozen rice sample was taken, 10 mL solution (70% (*v*/*v*) chloroform and 30% (*v*/*v*) methanol) was added, it was grinded into homogenate under liquid N condition, free amino acid was extracted with 8 mL of water, the water layer was extracted after 3 times, it was let to stand on ice for 30 min, then 500 μL supernatant was added and absorbed. The supernatant was filtered through a 0.45-μm micropore filter and loaded into a sample vial. Then, the automatic pre-column derivatization were used and single free amino acid concentrations were measured using Agilent 1260 High Performance Liquid Chromatography (Palo Alto, CA, USA).

### 4.6. Determination of the ^15^N-NH_4_^+^ Influx Rate

Rice seedlings were planted in IRRI solution containing 2.5 mM NH_4_^+^ for 2 weeks, then they were deprived of N for 3 days. Next, plants were first transferred into 0.1 mM CaSO_4_ for 1 min, then to a complete nutrient solution containing 2.5 mM ^15^NH_4_^+^ ((^15^NH_4_)_2_SO_4_) for 5 min, and finally to 0.1 mM CaSO_4_ for 1 min. Then, we used paper to blot the water on the plants. The shoots and roots were separated, then the samples were placed in an oven at 105 °C for 30 min to inactivate the enzymes, and further dried to a constant weight at 70 °C. After recording their dry weights, the samples were ground into powder using a ball mill. The Isotope Ratio Mass Spectrometer system (Thermo Fisher Scientific, Waltham, MA, USA) was used to determine the ^15^N content of the samples.

### 4.7. RNA In Situ Hybridization

Longitudinal sections of the rhizome junction of WT and *asn1* mutant seedlings with a length of about 5 mm were fixed in FAA solution (1.85% (*v*/*v*) formaldehyde, 5% (*v*/*v*) acetic acid, and 63% (*v*/*v*) ethanol), dehydrated with a mixture of ethanol and 1-butanol, and then embedded in paraffin as previously described. The embedded sections were sliced (10 μm in thickness) using a microtome (LEICA RM2235). A 500 bp cDNA fragment of *OsASN1* was amplified using gene-specific primers (F 5′-3′: CACCCAACCACAAGAAGATCAGGA; R5′-3′: TGCATGAGCCTCTTGATGAC) and cloned into pENTR-D-TOPO. Digoxin (DIG)-labeled RNA probes in sense or antisense orientation were synthesized using SP6 or T7 RNA polymerase, each linearized plasmid DNA was used as a template, using the DIG RNA labeling kit described in [8]. RNA in situ hybridization with DIG-labeled RNA probes as previously described [8,13] was done. And images was observed using the OLYMPUS BX51 optical microscope system (Tokyo, Japan) with OLYMPUS DP80 camera (Tokyo, Japan) and cellSens Standard imaging software (Tokyo, Japan).

### 4.8. Statistical Analysis

Data analysis processing and analysis of variance were performed using Microsoft Excel 2007 and STST ANOVA.

## Supplementary Materials

Supplementary materials can be found at http://www.mdpi.com/1422-0067/20/1/130/s1.
