# Supplementary material for: OsASN1 Plays a Critical Role in Asparagine-Dependent Rice Development
Source: Int J Mol Sci. 2018 Dec 31;20(1):130. doi: 10.3390/ijms20010130 (PMC6337572; doi:10.3390/ijms20010130)
Supplement: Supplementary file 1 [file ijms-20-00130-s001.pdf]

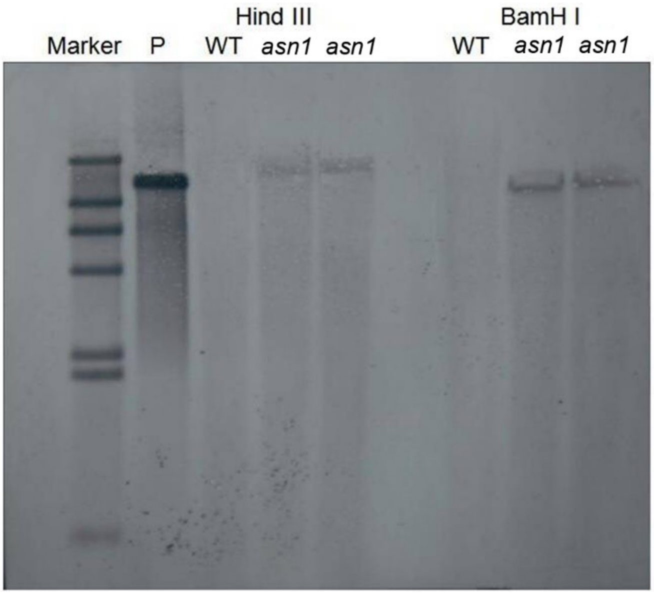

Figure S1. Detection of copy number of T-DNA in *asn1* by southern blot. DNA were digested by restriction enzyme Hind III and BamH I. P: positive control using plasmid.

Table S1. Primers used for QRT-PCR.

Gene Name	Gene ID	Primer Sequence（5' to 3'）
ACTIN	*Os03g0718100*	F:CTATGTTCCCTGGCATTGCT<br>R:TTTCCTGTGCACAATGGATG
ASN1	*Os03g0291500*	F: ACCGCATGATATTTGAGAGG<br>R: CTGCTCCACCCACTCGAT
ASN2	*Os06g0265000*	F:CGCTAGCTTTGTTTACCCAGA<br>R:CGACCAGATGGATCAAGGTT
GS1.1	*Os02g0735200*	F: CAAGTCTTTTGGGCGTGATATTGTTGAC<br>R:CACCTGATCACCGGCAGAAATGCCGACA
GS1.2	*Os03g0223400*	F:AAAGGCGTTCGGCCGCGACATCGTGGAC<br>R:CACTTGGTCAGCAGCGGCGATGCCAACT
GS2	*Os02g0701300*	F: AGAACTTGGACGATGAATCGGGGC<br>R: GAGGGAAGGACGCAGGACTGAAGA
NADH-GOGAT1	*Os01g0681900*	F:GTGCAGCCTGTTGCAGCATAAA<br>R:CGGCATTTCACCATGCAAATC
NADH-GOGAT2	*Os05g0555600*	F:GAACGGAAGGTTCCAATTCA<br>R: TGGTCGCAAAGTTTCCAAAT
Fd-GOGAT	*Os07g0658400*	F:GCATACTTGTGAAGCACCGAAGTG<br>R:CTGCAAATAGCAACCTAGCGTCAG
Asp AT1	*Os01g0760600*	F:GTGATCAAGTGGCTTTCATG<br>R:GTTTTGTGACTGCGGCAT
Asp AT2	*Os02g0797500*	F:CAGGGCGTATCAAGAATGTA<br>R:TTCTCCCATCCTTTGTCATG
Asp AT3	*Os02g0236000*	F:TGGATCGCTTAACCAATGAA<br>R:TCGTTTTAGTGACCTCATGA
Asp AT4	*Os06g0548000*	F:ATCTACATGACTCGTAACGG<br>R:ACTTTGTCTTGGTAACCTCA
Asp GB1	*Os04g0682500*	F:GAACTTGTTGCTGCCTATTC<br>R:AGTTGACACGAGTAGCAAAA
Asp GC1	*Os04g0549300*	F:GATGATGAAGTTGGTGCATG<br>R:AATATAGCATCCATGGCAGC
AGT1	*Os08g0502700*	F:CAAGGTGTTCAGGATCGG<br>R:GATGTCCTTGAGCACCATC
